# Comparisons of three anterior cervical surgeries in treating cervical spondylotic myelopathy

**DOI:** 10.1186/1471-2474-15-233

**Published:** 2014-07-10

**Authors:** RuoFu Zhu, HuiLin Yang, ZhiDong Wang, GenLin Wang, MinJie Shen, Quan Yuan

**Affiliations:** 1 Department of Orthopaedic Surgery, The First Affiliated Hospital of Soochow University, 188 Shizi Street, Suzhou, China

**Keywords:** Cervical spondylotic myelopathy, Fusion, Non-fusion, Dynamic cervical implant, Clinical effects

## Abstract

**Background:**

Anterior cervical discectomy and fusion (ACDF) was one of the preferred treatments for degenerative cervical spondylosis. However, the motion of adjacent segment was significantly increased after operation. So cervical disc arthroplasty have been suggested to keep the motion of adjacent segment. A new implant named dynamic cervical implant (DCI) has been developed to keep the motion of adjacent segment.

**Methods:**

We retrospectively reviewed 91 patients treated for single level cervical spondylotic myelopathy with anterior cervical discectomy and fusion (ACDF), dynamic cervical implant (DCI) and cervical total disc replacement (CTDR) between sep 2009 and Mar 2011 in our hospital. They were divided into three groups by surgical methods: ACDF group (group A, 34 cases), DCI group (group B, 25 cases), CTDR group (group C, 32 cases). Operation time, intraoperative blood loss, preoperative and postoperative JOA score and JOA recovery rate were compared among the three groups. Pre-and postoperative hyperextension and hyperflexion radiograms were observed to measure range of motion (ROM) of C_2–7_, operative and adjacent levels.

**Results:**

There was no statistical difference in operative time, intraoperative blood loss, and JOA recovery rate (*P* > 0.05) among three groups. But the differences of their postoperative JOA scores and preoperative JOA scores were of statistical significance (*P* < 0.05). Compared the pre-and postoperative ROM of C_2–7_, operative, upper and lower levels of each group respectively, the difference between preoperative ROM and postoperative ROM of group A were of statistically significant (*P* < 0.05), while was no statistically significant of group C (*P* > 0.05). There was no statistically significant difference between preoperative ROM and postoperative ROM of upper and lower levels in group B (*P* > 0.05), but had statistically significance of C_2–7_ and operative levels (*P* < 0.05).

**Conclusions:**

Three operations are effective therapies for single level cervical spondylotic myelopathy. But each group has respective advantages and disadvantages.

## Background

Since 1950s, anterior cervical discectomy and fusion (ACDF) has been considered as the golden standard of treating middle-aged degenerative cervical spondylosis [1,2]. But as is found in long-term clinical researches, the motion of cervical spine might decrease after ACDF, while fused adjacent segments accelerate degeneration [3-5]. Cervical total disc replacement can maintain the motion of surgical segments as well as the cervical stability, however, it has a high incidence of Heterotopic Ossification and kyphosis with few indications [6]. Dynamic cervical implant (DCI) is a U-shaped titanium alloy stabilizing device for cervical implantation recently designed by GCI, which provides cervical dynamic stability under non-fusion. This research compares the curative effects and ROM of C_2–7_, operative, upper and lower levels of three anterior approaches in treating single level cervical spondylotic myelopathy.

## Methods

91 cases of single level cervical spondylotic myelopathy diagnosed and followed up in our hospital from September 2009 to March 2011 were conducted among people who underwent the examination of anteroposterior, lateral, hyperextension and hyperflexion X-rays, CT scan and MRI. Among them, 34 cases underwent anterior cervical discectomy and fusion with traditional titanium plate and Cage (Group A), and the devices were ATLANTIS titanium plate and PEEK CornerStone-SR cage provided by Medtronic. There were 16 females and 18 males with an average age of 49.54 years (range 32–69 y) at surgery. The average duration of symptoms was 10.75 months (range 1–55 months). Postoperative follow-up was 13–32 months, with an average time of 20.33 months. C_3–4_ 2 cases, C_4–5_ 9 cases, C_5–6_ 14 cases, C_6–7_ 9 cases. 25 cases underwent DCI implantation (Group B), and the devices were the DCI of GCI. There were 11 females and 14 males with an average age of 47.07 years (range 23-62y) at surgery. The average duration of symptoms was 11.13 months (range 2–50 months). Postoperative follow-up was 14–33 months with an average follow-up was 19.93 months. C_3–4_ 2 cases, C_4–5_ 7 cases, C_5–6_ 11 cases, C_6–7_ 5 cases. 32 cases underwent cervical total disc replacement with prestige prosthesis (Group C), and the device was provided by Medtronic. There were 12 females and 10 males with an average age of 48.00 years (range 34-67 y) at surgery. The average duration of symptoms was 10.63 months (range 2-60 months). Postoperative follow-up was 12–30 months with an average follow-up of 20.18 months. C_3–4_ 1 cases, C_4–5_ 6 cases, C_5–6_ 15 cases, C_6–7_ 10 cases. There was no statistical difference in age, follow-up time and duration of symptoms among the three groups (*P* > 0.05). This study was approved by the Institutional Ethics Committee of Soochow University.

### Surgical procedure

All surgeries were conducted by a chief physician of our hospital. After cervical plexus anesthesia or endotracheal intubation for general anaesthesia, the patients were placed with ahead extension in supine position. **Group A:** The operations were performed using standard anterior cervical discectomy and fusion. **Group B and C:** A transverse incision is made in a skin crease at the appropriate disc level on the right side of the neck. After confirmation and exposure of the appropriate vertebral levels. The Casper distracter was placed and the disc materials were removed and the resection of posterior longitudinal ligament was applied depending on the pressurized severity of dural sac. **Group B**: After decompression, model testing was conducted with DCI specific tool under fluoroscopy, placing corresponding DCI model in intervertebral space. The insertion depth was 2-3 cm away from the vertebral body. As the position was observed to be satisfying under fluoroscopy with C-arm X-ray machine, and then conventionally close the incision. **Group C**: After decompression, we put the tested models into intervertebral space to get appropriate prosthesis, and used different models of files to polish the endplate as flat as possible and keep its cortical bone, to get filing marks on the bone plate, as well as appropriate intervertebral space. Guided by pilot sleeve, gently knock out a groove separately on upper and lower endplates for prosthesis implantation, implanted the prestige prosthesis into intervertebral space. As the position was observed to be satisfying under fluoroscopy with C-arm X-ray machine, and then conventionally close the incision. The operation time and the intraoperative blood loss for the three groups were recorded.

### Clinical outcome assessment

Follow-up clinical examinations were obtained by a physician unrelated to the surgical procedures. The clinical outcomes were evaluated using Japanese Orthopaedic Association (JOA) score before and after operations. A recovery rate (RR) was also calculated, which was defined according to the rationale of Hirabayashi et al. [7] as RR = (postoperative JOA scores- preoperative JOA scores)/(17- preoperative JOA scores) × 100%. Results were indicated by the RR as follows: 75% or more (excellent), 50% to 74% (good), 25% to 49% (fair), and less than 25% (poor). The patients’ conditions were divided into three levels and indicated by the JOA scores as follows: less than 7 score (severe), 8 score to 12 score (moderate), 13 score to 16 scores (Mild).

### Radiological evaluation

All follow-up patients underwent anteroposterior, lateral, hyperextension and hyperflexion X-rays, the range of motion (ROM) of C_2–7_, operative and adjacent levels were measured on hyperextension and hyperflexion radiograms according to the Cobb’ method. The imaging data were separately and independently measured blindedly by three orthopaedics physician, the results were mean value.

### Statistical analysis

A Paired- samples *t* test was used for the paired data and an One-Way ANOVA was used for Three-samples data. The two-tailed test ’s results were considered significant when *P* was less than 0.05. All the analyses were performed using Microsoft Excel 2003 (Microsoft, Seattle, WA) and SPSS 16.0 (SPSS, Chicago, IL,USA).

## Results

There was no infection, hematoma, hoarseness or blood loss in three groups, or hematoma, infection, fracture or chronic pain in bone harvesting area of iliac crest. In group A, there were three cases with postoperative dysphagia in one week and recovered in two months. There was no statistical difference in operative time, intraoperative blood loss, and JOA recovery rate (*P* > 0.05) among three groups, But postoperative JOA scores differ significantly from their preoperative JOA scores (*P*_A_ = 0.000, *P*_B_ = 0.000, *P* < 0.01) (Table 1). The JOA recovery rate in group A were calculated as follows: 12.5% (excellent), 70.8% (good), 12.5 (fair), and 4.1% (poor). The JOA recovery rate in group B were calculated as follows: 20.0% (excellent), 66.7% (good), 13.3 (fair). The JOA recovery rate in group C were calculated as follows: 13.6% (excellent), 72.7% (good), 13.6% (fair).

**Table 1:** Comparisons of intraoperative blood loss, operative time and JOA scores among three groups

	**Intraoperative blood loss**	**Operative time**	**Preoperative JOA score**	**Postoperative JOA score**	**JOA recovey rate**
Group A	94.79 ± 14.33	109.79 ± 18.97	9.54 ± 0.88*	13.96 ± 1.52*	0.60 ± 0.18
Group B	93.33 ± 13.05	125.00 ± 18.13	9.33 ± 1.18*	14.07 ± 1.79*	0.62 ± 0.21
Group C	91.59 ± 15.77	116.59 ± 20.49	9.27 ± 0.83*	14.00 ± 1.19*	0.62 ± 0.16
F value	0.277	2.871	0.506	0.024	0.053
P value	0.759	0.065	0.605	0.976	0.949

Means ± standard deviation.

*The differences of their postoperative JOA scores and preoperative JOA scores were of statistical significance (*P*_A_ = 0.000, *P*_B_ = 0.000, *P*_C_ = 0.000, *P* < 0.01).

**Figure 1:**
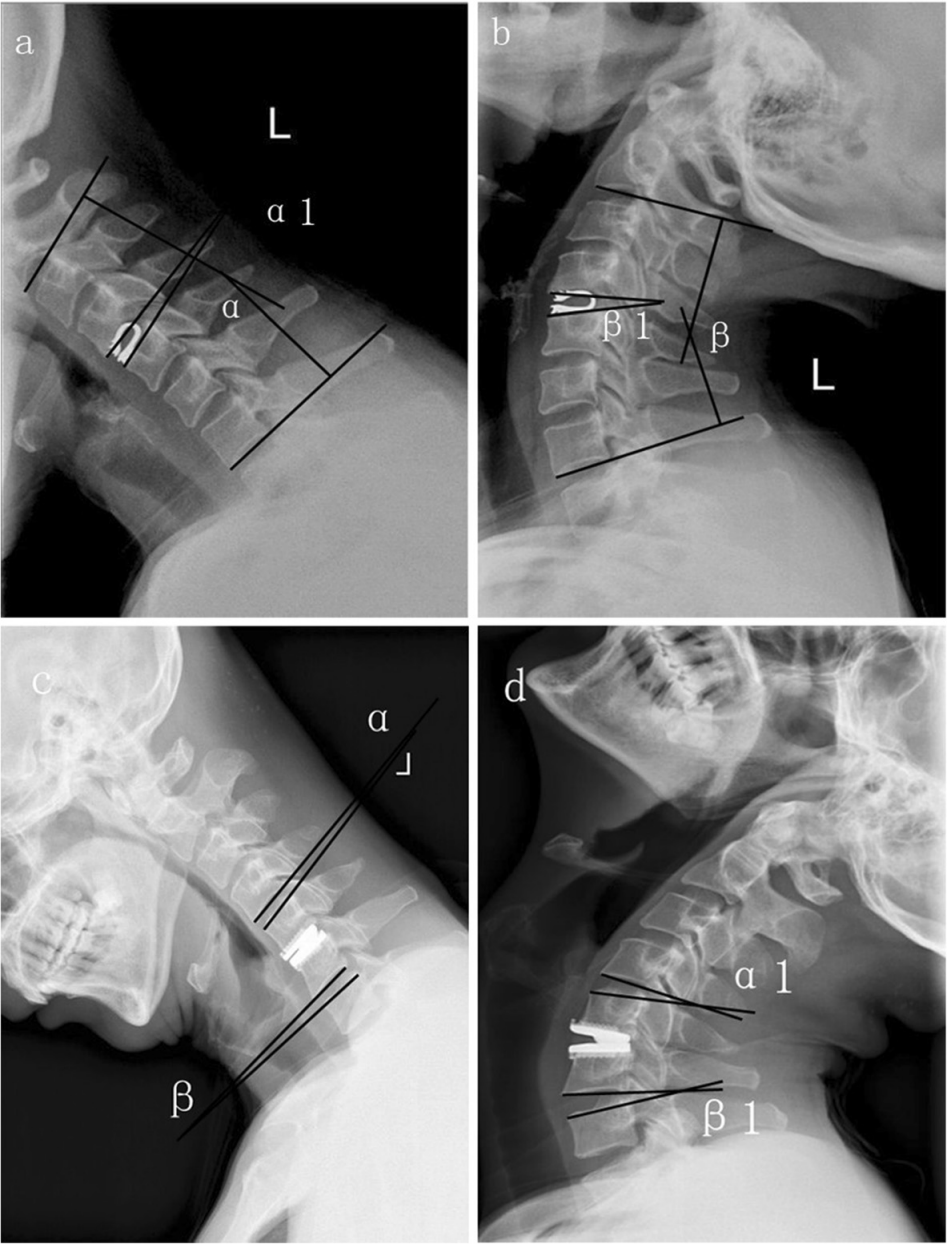
**Hyperextension and hyperflexion radiograms of postoperative DCI and CTDR. a**, **b** angle α plus angle β is ROM of C_2–7_, angle α1 plus angle β1 is ROM of operative level. **c**, **d** angle α plus angle α1 is ROM of upper level, angle β plus angle β1 is ROM of lower level.

Postoperative cervical anteroposterior and lateral X-rays indicate that positions of internal fixtion are in good state, without loosening, displacement or rupture in the three groups. Hyperextension and hyperflexion lateral radiographs measure of C_2–7_ ROM, ROM of surgical segments, ROM of upper adjacent segments and lower adjacent segments (Table 2). Figure 1 displayed measure of ROM of C2-7, surgical segments, adjacent segments upper and lower levels.

**Table 2:** **Sagittal ROM of C**_**2-7**_**, implanted level and adjacent levels**

	**Group A**	**Group B**	**Group C**
C_2–7_ ROM			
Pre-op	49.92 ± 8.17	49.13 ± 8.04	49.18 ± 7.82
Post-op	41.08 ± 4.74	44.73 ± 6.90	48.59 ± 6.80
P value	< 0.001	< 0.001	0.091
Implanted level ROM			
Pre-op	8.88 ± 1.33	9.20 ± 1.26	8.91 ± 1.48
Post-op	0	7.13 ± 1.19	8.59 ± 1.68
P value	< 0.001	< 0.001	0.216
Upper level ROM			
Pre-op	8.83 ± 1.83	8.87 ± 1.46	9.05 ± 1.86
Post-op	10.75 ± 1.82	9.33 ± 2.72	9.23 ± 2.52
P value	< 0.001	0.543	0.728
Lower level ROM			
Pre-op	9.04 ± 1.46	8.93 ± 1.90	9.27 ± 1.75
Post-op	11.08 ± 1.56	9.80 ± 2.51	9.45 ± 2.22
P value	< 0.001	0.138	0.683

Means ± standard deviation.

## Discussion

Cervical spondylotic myelopathy is a common disease endangering human beings. When the patients have failed formal conservative treatment and worsened syndromes, he should undergo surgery. All surgical treatments achieve effects by discectomy for relieving spinal cord compression, and what differs is the approach which is typical fusion or cervical arthroplasty that emphasizes on maintaining the motion of surgical segments in recent years. In 1958, Robinson, Smith [8] and Cloward [9] successively adopted ACDF to treat cervical spondylosis caused by intervertebral disc degeneration with good clinical effects, so it became the typical surgical method to treat cervical spondylosis. However, as is found in numerous long-term clinical cases, ACDF has some complications like reduced cervical activity and accelerated degeneration of adjacent-segment disc [10]. After as long as 21 years follow-up of 374 cases undergoing ACDF, Hilibrand et al. [11] found that the clinical syndromes caused by fusion adjacent segment degeneration had an average incidence of 2.9% in postoperative 10 years, which requires clinical focus and in-depth research.

Because of the above-mentioned complications, the cervical arthroplasty focusing on maintaining surgical segments’ motion is clinically applied. Cervical total disc replacement can keep cervical motion and decrease the incidence of adjacent segments’ degeneration with excellent clinical effects in early and middle periods [12,13]. However, substantial follow-ups found that it has a high incidence of kyphosis and Heterotopic Ossification [6,14]. The design idea of cervical total disc replacement is to imitate joint prosthesis, but the movements of spinal multi-segments is more complex than that of hip and knee joints, and the influencing factors are numerous, while the implantation of prosthesis requires greatly, such as the selection of prosthesis size, the treatment of upper and lower endplates of replaced segments, the height of prosthetic joint line, rotating axis, position of rotating center and so on, which are still lack of good explanation. In the literature and reports, there are a great many complications in and after surgery [15,16]. Unlike hip and knee joint replacement, the researches into cervical disc replacement is not thorough enough to replace fusion surgery, so it requires further study. DCI is another try of cervical non-fusion technology. Being similar to cervical disc replacement surgery, it is also the analog reconstruction of some features and characteristic of restoring the intervertebral disc height and maintaining certain stability. DCI’s dynamic design has axial compliance and shock absorption, effectively avoiding the accelerated degeneration of upper and lower adjacent segments. DCI possesses three alternatives of height and 4 models (Table 3), restoring and maintaining intervertebral height and being suitable for all endplates.

**Table 3:** Dynamic cervical implant size (mm)

**Height**	**Size (length × width)**			
	**S**	**M**	**L**	**XL**
5	10 × 12	12 × 14	14 × 16	16 × 18
6	10 × 12	12 × 14	14 × 16	16 × 18
7	10 × 12	12 × 14	14 × 16	16 × 18

**Figure 2:**
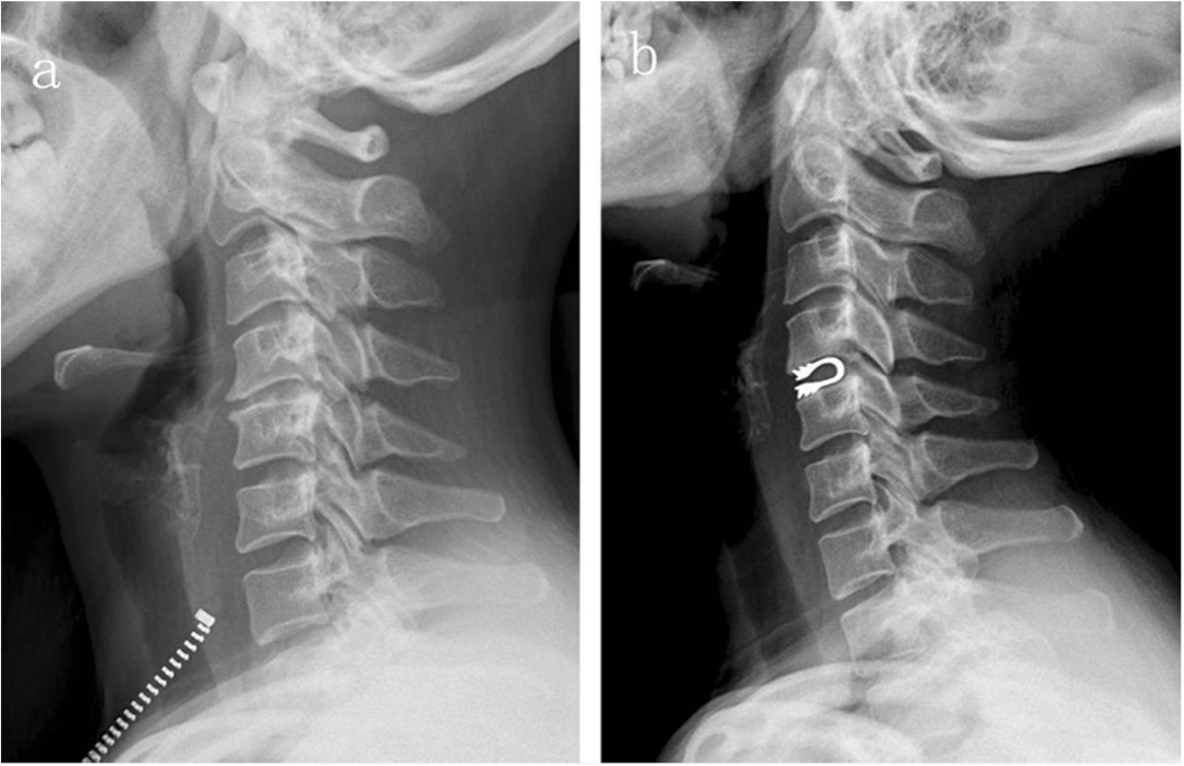
**Preoperation and postoperation of DCI. a** Preoperative lateral X-rays showed the height of C4-5 decreased, and kyphosis existed. **b** Postoperative lateral X-rays showed the kyphotic curvature retrieved.

There was no statistical difference in operative time, intraoperative blood loss, and JOA recovery rate (*P* > 0.05) among three groups. It proves that new DCI has the same early clinical effect with anterior fusion and cervical disc replacement. However, the patients undergoing cervical disc replacement or DCI have maintained cervical stability and activity to some degree, which contributes the life quality improvement. Group A has a postoperative ROM of C_2–7_ of 41.08° ± 4.74°, reducing by 8.83° ± 5.53° when compared with the preoperative one, and the difference is of statistical significance (*P* < 0.01). After fusion, the cervical motion segment number decreases, causing cervical motion reduces. All ROM of surgical segments had bony fusion after surgery with the activity of 0°. Compared with the preoperative one, the difference is of statistical significance (*P* < 0.01); postoperative ROM of upper adjacent segment is 10.75° ± 1.82°, having a rise of 1.92° ± 1.25° as compared with the preoperative one, and the difference is of statistical significance (*P* < 0.01); the ROM of lower adjacent segment is 11.08° ± 1.56°, having a rise of 2.04° ± 1.30° as compared with the preoperative one, and the difference is of statistical significance (*P* < 0.01). After fusion, the movement of adjacent segments increases, leading to the degeneration of adjacent segments, which is consistent with the report of Sasso et al. [10]. Group B has a postoperative ROM of C_2–7_ of 44.73° ± 6.90°, reducing by 4.40° ± 2.47° as compared with the preoperative one, while the postoperative ROM of surgical segments is 7.13° ± 1.19°, reducing by 2.07° ± 1.22°, as compared with the preoperative one, and the differences are of statistical significance (*P* < 0.01). It is also caused by the semi-restrictive activity of DCI, which keeps ROM of surgical segments as well as prevents excursive movement of cervical posterior facet joints, so as to benefit protecting the posterior stability. The postoperative ROMs of upper and lower adjacent segments are 9.33° ± 2.72° and 9.80° ± 2.51°, respectively. Compared with the preoperative ones, the differences are of no statistical significance (*P* > 0.05), which manifests that after surgical segments of DCI, ROM of adjacent segments does not increase, so it can reduce degradation of adjacent segments. Meanwhile, the follow-up found that 3 cases of cervical deformity could be improved obviously after DCI implantation, so DCI implantation can improve the cervical kyphosis to some degree (Figure 2). Group C has a postoperative ROM of C_2–7_ of 48.59° ± 6.80°, and postoperative ROM of surgical segment is 8.59° ± 1.68°, upper adjacent segment 9.23° ± 2.52° and lower adjacent segment 9.45° ± 2.22°. Compared with the preoperative ones, the difference were of no statistical difference (*P* > 0.05). It is because cervical artificial disc is unlimited, which can maintains the motion of surgical segments and do not influence cervical integral movement. When compared with unlimited cervical artificial disc, whether the semi-restrictive DCI’s protection of posterior small facet joints and the influence on daily life is more good than harmful requires further research.

## Conclusions

The three anterior cervical surgical approaches have good curative effects on single level cervical spondylotic myelopathy. Both anterior fusion and cervical total disc replacement have their own advantages and disadvantages, and DCI implantation, as a new non-fusion approach, is easy and simple to operate with short learning curve. It is an interbody fixed device between ACDF and cervical disc replacement surgery, being able to partially keep the motion function of cervical surgical segments and an new alternative of treating cervical spondylosis. Although its short-term effect is satisfactory, it has a short follow-up time and few cases. Whether DCI prosthesis will be loose, fall off or submerge, whether heterotopic Ossification occurs around DCI prosthesis and what are the middle and long-term curative effects require a long follow-up.

## Competing interests

The authors have indicated that they have no conflict of interest regarding the content of this report.

## Authors’ contributions

All authors made substantive intellectual contributions to this study to qualify as authors. RZ, ZW and HY contributed to study design, acquisition of data, analysis of data, and interpretation of results. GW contributed to study coordination. MS and QY contributed to statistical analysis. RZ, ZW, HY and GW contributed to manuscript preparation. All authors read and approved the final manuscript.

## Pre-publication history

The pre-publication history for this paper can be accessed here:

http://www.biomedcentral.com/1471-2474/15/233/prepub
